# Sodium Tanshinone II-A Sulfonate (DS-201) Induces Vasorelaxation of Rat Mesenteric Arteries via Inhibition of L-Type Ca^2+^ Channel

**DOI:** 10.3389/fphar.2018.00062

**Published:** 2018-02-02

**Authors:** Xiao-Dong Zhang, Chun-Xia He, Jun Cheng, Jing Wen, Peng-Yun Li, Na Wang, Guang Li, Xiao-Rong Zeng, Ji-Min Cao, Yan Yang

**Affiliations:** Key Laboratory of Medical Electrophysiology of Ministry of Education, Collaborative Innovation Center for Prevention and Treatment of Cardiovascular Disease, Institute of Cardiovascular Research, Southwest Medical University, Luzhou, China

**Keywords:** sodium tanshinone II-A sulfonate (DS-201), large conductance Ca^2+^-activated K^+^ channel (BK_Ca_ channel), L-type calcium channel, calcium dynamics, blood vessel

## Abstract

**Background:** We previously have proved that sodium tanshinone II-A sulfonate (DS-201), a derivative of traditional Chinese medicinal herb Danshen (Salvia miltiorrhiza), is an opener and vasodilator of BK_Ca_ channel in the vascular smooth muscle cells (VSMCs). Vascular tension is closely associated with Ca^2+^ dynamics and activation of BK_Ca_ channel may not be the sole mechanism for the relaxation of the vascular tension by DS-201. Therefore, we hypothesized that the vasorelaxing effect of DS-20 may be also related to Ca^2+^ channel and cytoplasmic Ca^2+^ level in the VSMCs.

**Methods:** Arterial tension was measured by Danish Myo Technology (DMT) myograph system in the mesentery vessels of rats, intracellular Ca^2+^ level by fluorescence imaging system in the VSMCs of rats, and L-type Ca^2+^ current by patch clamp technique in Ca^2+^ channels transfected human embryonic kidney 293 (HEK-293) cells.

**Results:** DS-201 relaxed the endothelium-denuded artery rings pre-constricted with PE or high K^+^ and the vasorelaxation was reversible. Blockade of K^+^ channel did not totally block the effect of DS-201 on vasorelaxation. DS-201 suppressed [Ca^2+^]_i_ transient induced by high K^+^ in a concentration-dependent manner in the VSMCs, including the amplitude of Ca^2+^ transient, the time for Ca^2+^ transient reaching to the [Ca^2+^]_i_ peak and the time to remove Ca^2+^ from the cytoplasm. DS-201 inhibited L-type Ca^2+^ channel with an EC_50_ of 59.5 μM and at about 40% efficacy of inhibition. However, DS-201did not significantly affect the kinetics of Ca^2+^ channel. The effect of DS-201 on L-type Ca^2+^ channel was rate-independent.

**Conclusion:** The effect of DS-201 on vasorelaxation was not only via activating BK_Ca_ channel, but also blocking Ca^2+^ channel and inhibiting Ca^2+^ influx in the VSMCs of rats. The results favor the use of DS-201 and Danshen in the treatment of cardiovascular diseases clinically.

## Introduction

Danshen (*Salvia miltiorrhiza*), a traditional Chinese medicinal herb, is effective in the prevention and treatment of various cardiovascular diseases including angina pectoris, hyperlipidemia and acute ischemic stroke (Valli and Giardina, 2002; Zhou et al., 2005; Cheng, 2006; Chan et al., 2009). Tanshinone II-A is a diterpene quinine and the main active derivative of Danshen. DS-201, a water-soluble derivative of tanshinone II-A, is suitable for clinical administration. DS-201 is effective in suppressing atherosclerosis, reducing myocardial infarct size, and increasing coronary blood flow and myocardial contractility (Cheng, 2007). DS-201 is currently used for clinical treatment of angina pectoris, myocardial infarction, and thrombosis in the cerebral artery, central retinal artery and peripheral vein. However, the underlying mechanisms of DS-201 including how to relax the vascular tension are still not well clarified.

The activity of K^+^ channel in the VSMCs determines the levels of resting membrane potential and action potential repolarization and hyperpolarization for causing a buffering mechanism to counteract membrane potential depolarization and vascular constriction. Large conductance Ca^2+^-activated K^+^ (BK_Ca_) channel is the main K^+^ channel in blood vessel for carrying 60-70% of the outward currents, thus it plays a pivotal role in vascular relaxation (Jaggar et al., 2000; Wray et al., 2005). We previously reported that DS-201 induced vasodilatation via activating the BK_Ca_ channel in a concentration-dependent manner (Yang et al., 2008; Tan et al., 2011; Yu et al., 2016). We also found that DS-201 at high concentration (more than 100 μM) decreased BK_Ca_ currents, especially STOCs, suggesting that Ca^2+^-associated action may play a role in the process of BK_Ca_ activity. It is known that activation of BK_Ca_ channel is associated with vasodilation, and then the inhibition of BK_Ca_ channel by high concentrations of DS-201 should counteract its vasorelaxing effect. However, DS-201 at high concentrations still possessed the effect of vasorelaxation. This phenomenon suggests that an alternative mechanism may exist for DS-201 in vasorelaxation. It is well known that Ca^2+^-associated signaling is an important determinant of vascular tone. Vascular constriction and relaxation depend on the cytosolic free Ca^2+^ level ([Ca^2+^]_i_) which can come from either Ca^2+^ influx through L-type Ca^2+^ channel in the plasma membrane or receptor-mediated Ca^2+^ release from the intracellular Ca^2+^ stores including the sarcoplasmic reticulum (SR). Danshen and its derivatives were reported to have beneficial effects on stroke and ischemic diseases because of their properties of vasodilation and hypotension. For example, Lam et al (Lam et al., 2006) reported that the vasorelaxing action of Danshen and its fractions was produced primarily through inhibition of Ca^2+^ influx and only a small component was mediated by opening of K^+^ channel in the VSMCs. The same group also found that dihydrotanshinone, a lipophilic component of Danshen, could relax coronary artery by inhibition of Ca^2+^ channel in rat (Lam et al., 2008). However, question remains whether DS-201 (a derivative of Danshen) could also affect Ca^2+^ influx and thus affect vascular tone in the VSMCs? The present study was focused on a possible new mechanism of vasodilatation induced by DS-201.

## Materials and Methods

### Chemicals

DS-201 (98% purity) was obtained from the National Institutes for Food and Drug Control (NIFDC, Beijing, China). PE, ACh, TEA, IbTX, Bay K 8644 and Nifedipine were purchased from Sigma–Aldrich Inc. (St. Louis, MO, United States). Fura-2 AM (5-Oxazolecarboxylic acid, 2-(6-(bis(2-((acetyloxy)methoxy)-2-oxoethyl)amino) -5-(2-(2-(bis(2-((acetyloxy)methoxy)-2-oxoet-hyl)amino)-5-methylphenoxy)ethoxy)-2-benzofuranyl)-, (acet-yloxy)methyl ester) was purchased from Invitrogen Inc. (San Diego, CA, United States).

### Cell Culture and Transfection

Human embryonic kidney 293 (HEK293) cells were transiently transfected with the smooth muscle predominant CaV1.2 channel isoform Cav1.2SM (1/8/9^∗^/32/Δ33) plus the subunits of β2a and α2δ and cultured in modified RPMI-1640 medium containing 10 % fetal bovine serum (FBS) and 1% Penicillin–Streptomycin solution at 37°C and 5% CO_2_.

### Experimental Animals

Six-month old specific-pathogen-free (SPF) male Sprague–Dawley (SD) rats (250–300 g) were obtained from the Animal Care Center of Southwest Medical University (Luzhou, Sichuan, China). The rats were housed up to four rats per cage with free access to water and food at a constant room temperature (∼25°C) in a 12-h light/12-h dark cycle. All animal experiments were performed strictly in accordance with university guidelines and an approved animal study protocol by the Committee on Use and Care of Animals of Southwest Medical University (Luzhou, Sichuan, China).

### Measurement of Arterial Tension

Rats were anesthetized with 1% pentobarbital sodium (50 mg/kg) and the mesenteric arteries were isolated and the artery rings were used for the measurement of arterial tension. Briefly, artery rings (2–3 mm long) were quickly obtained from the secondary and tertiary branches of the mesenteries in rats under a binocular microscope and placed in ice-cold normal Tyrode’s solution (in mM: NaCl 127.0, KCl 5.9, MgCl_2_ 1.2, CaCl_2_ 2.4, Na-HEPES 10.0, glucose 12.0, pH 7.4). The VSMCs from each vessel used in the experiments were first detected the presence of endothelial cells and removed them in order to exclude the effect of endothelial cells. The endothelial cells were removed by the method of 0.1% Triton-100 perfusion after comparison of the mechanical method (fine wire slide into the lumen of the blood vessel) to prove that it was easier to be controlled with more stable effect. The artery rings were mounted in a Danish Myo Technology (DMT) myograph under a normalized tension after removal of the endothelial cells as previously described (Yang et al., 2013). The resting tension of the artery rings was adjusted according to the guide of the data acquisition system and balanced for 1 h before vasomotor experiments. Briefly, arterial rings were stretched in a step-wise manner and set to 0.9× IC100 (the internal circumference equivalent to a transmural pressure of 100 mmHg) to determine the optimal resting tension. Equimolar KCl was used to replace NaCl in Tyrode solution to prepare 60 mM KCl solution (high K^+^ solution, adjust pH to 7.4 with NaOH). The maximal vasoconstriction was detected by high K^+^ solution after 1 h balance. One micromole ACh was added to detect endothelial cells when the vasocontraction reached the maximum and stable state. The arteries with less than 10% relaxation induced by ACh were used for subsequent experiments. The gradient concentrations for the maximal response of artery rings to PE were tested and determined 3 μM as the optimal concentration, thus it was selected for subsequent experiments. Vascular responses to DS-201 (20 to 200 μM) were observed following preconstriction with PE (3 μM) and high K^+^ solution. The vasoreactivity of DS-201 was also investigated by incubating the artery rings with 5 mM TEA or 200 nM IbTX (a BK_Ca_ channel blocker) for 10 min. The maximal contraction induced by PE and high K^+^ solution was defined as 100%. The percentage of relaxation at each DS-201 concentration was used to draw the concentration-response curve and the curve was fitted with the dose-response function to obtain the half maximal effective concentration (EC_50_). The X axes in the dose-response curves were log transformed in such cases and the curves were typically sigmoidal, with the steepest portion in the middle, so to visually imply a threshold concentration and EC_50_.

### Preparation of VSMCs of SD Rats

Single VSMC was enzymatically isolated from the mesentery arteries of SD rats as described previously (Yang et al., 2008). Briefly, mesentery arteries were obtained by removal of the surrounding tissues of the arteries under a microscope. Then the arteries were cut into 1-mm pieces and incubated in a Ca^2+^-free Tyrode’s solution containing in mg/mL: 1.0 papain, 2.0 albumin, and 2.0 dithiothreitol (DTT) for 8–10 min, followed by a fresh Ca^2+^-free Tyrode’s solution containing 1.25 mg/mL collagenase XI (Sigma–Aldrich, St. Louis, MO, United States) for 6-8 min at 37°C with gentle agitation. The isolated VSMCs were kept in 0.1 mM [Ca^2+^] Tyrode’s solution at 4°C, and were freshly used for the measurement of [Ca^2+^]_i_.

### Measurement of [Ca^2+^]_i_

Intracellular Ca^2+^ transients were measured with fura-2 fluorescence at room temperature (21 ± 2°C) by a dual excitation wavelength fluorescence method as described previously (Grynkiewicz et al., 1985; Wang et al., 2003) using the TILLvisION 4.0 imaging system (Till Photonics, Gräfelfing, Germany). Freshly isolated mesenteric VSMCs of rats were loaded with 5 μM fura-2/AM for 30 min. The dye was excited by alternatively using 340 nm (20 ms) and 380 nm wavelengths (10 ms) lights with a Xenon 75 W arc lamp. The emission fluorescence at 510 nm was detected by a photomultiplier tube. Photobleaching was minimized by the use of neutral density filters and shuttering excitation light (97 ms) during experiments. The intracellular free Ca^2+^ concentration ([Ca^2+^]_i_) was calculated using the following equation: *[Ca^2+^]_i_ = Kd ^∗^(Sf2/Sb2)^∗^ (R – Rmin)/(Rmax – R)*, where *Kd* as the dissociation constant for fura-2/calcium complex, *R* as the ratio of the emission fluorescence evoked by 340 and 380 nm light excitation, *Rmin* as the ratio obtained in the Ca^2+^-free Tyrode’s solution with 10 mM EGTA, *Rmax* as the ratio obtained in the saturating [Ca^2+^] solution (10 mM [Ca^2+^] Tyrode’s solution), and *Sf2/Sb2* as the ratio of emission fluorescence evoked by 380 nm excitation in Ca^2+^-free Tyrode’s solution and saturating [Ca^2+^] solution. A *Kd* value of 224 nM was used for the calculation. Ionomycin (10 μM) was added in the solution for the measurement of the values of *Rmax* and *Rmin*.

High K^+^-induced Ca^2+^ transients in the VSMCs of rats were obtained by applying of 60 mM high K^+^ solution for 10 s using a drug delivery system (ALA VM4, ALA Scientific Instrument, Farmingdale, NY, United States). The effect of DS-201 on high K^+^-induced Ca^2+^ transients was observed after 10 min pre-incubation of the cells with DS-201 and then applied high K^+^ (60 mM) for 10 s. The cells were continuously washed out with Tyrode’s solution during the 10-min interval. High K^+^-evoked Ca^2+^ transient was presented as the change of [Ca^2+^]_i_ from the base level to the peak after the treatment of high K^+^ solution for 10 s. Ca^2+^ transient rise time was defined as the time from the base level to the peak of [Ca^2+^]_i_. Ca^2+^ transient decay time was defined as the time for 90% reduction from the peak of [Ca^2+^]_i_.

### Electrophysiology

Whole-cell voltage clamp recordings were conducted using an EPC-10 patch clamp amplifier and Pulse software (Heka Elektronik, Lambrecht, Germany). L-type Ca^2+^ (L_Ca_) channel-transfected HEK-293 cells were placed in a small chamber on an inverted microscope (IX71, Olympus, Japan) and perfused with bath solution. L-type Ca^2+^ current (*I*_Ca,L_) was measured with the whole-cell patch clamp technique. Voltage commands were given to elicit Ca^2+^ currents. The Ca^2+^ currents were measured 15 min after the formation of whole-cell configuration to allow equilibration between pipette solution and cytosole. The current capacity was measured for each cell during the 20-ms pulses from a holding potential of –80 mV to a testing potential of -85 mV. The capacity currents and residual leak currents were subtracted using P/5 protocol. The current–voltage (I–V) relationship was determined by 400 ms depolarizing pulses to potentials ranging from –50 mV to +50 mV from a holding potential of –80 mV in 10 mV increments at 0.1 Hz. The concentration- dependent relationship of drug on *I*_Ca,L_ was examined by measuring peak inward current for cell depolarized from –50 mV to +50 mV in the presence or absence of DS-201. The voltage-dependence of steady-state inactivation was determined by 4800 ms conditioning prepulses from –120 mV to +50 mV in 10 mV increments, followed by a test pulse of +30 mV for 300 ms. To measure the rate-dependent effect of DS-201, a 15-series depolarizing pulses with 400 ms duration from a holding potential of -80 mV to +10 mV at different stimulation frequencies (0.1, 0.2, 0.7, and 2.0 Hz) were applied without use of P/5 leak subtraction.

Membrane currents were filtered at 1.0 kHz and sampled at 10 kHz. Data were stored in a computer for offline data analysis. Current densities (pA/pF) were obtained for each cell to normalize the whole cell currents. For recording of Ca^2+^ channel current in whole-cell configuration, the bath solution was used with K^+^-free solution (in mM): NaCl 130, TEA-Cl 4, CsCl 1, BaCl_2_ 10, MgCl_2_ 1.2, D-glucose 10, and HEPES 10, pH adjusted to 7.4 with CsOH. The pipette solution contained (in mM): Cs-aspartic acid 115, CsCl 20, MgCl_2_ 2.5, EGTA 10, HEPES 10, and Na_2_ATP 2, pH adjusted to 7.2 with CsOH. The presence of Cs^+^ instead of K^+^ in the solution blocks the potassium currents. All experiments were performed at room temperature (20–22°C). The results from pre-experiment showed that the *I*_Ca,L_ within 15–40 min after the formation of whole-cell configuration was relatively stable (rundown <10%). Therefore, we measured the effect of DS-201 during this period.

### Statistical Analysis

The data and statistical analysis comply with the recommendations on experimental design and analysis in pharmacology (Curtis et al., 2015). All data were expressed as mean ± standard error (SE). Statistical differences were analyzed by IBM SPSS statistics software version 19 (IBM Corp, Chicago, IL, United States). For statistical comparisons, the data were first evaluated to see whether they were normally distributed. Then, the data were reexamined for similar variances among normally distributed data, followed by Student’s *t*-test for the comparisons between two-group and analysis of variance (ANOVA) for more than two groups if the evaluations of similar variances were passed. The significance between groups were determined by one-way ANOVA and student-Newman–Keuls test for the effects of DS-201 on vasorelaxation, Ca^2+^ transients, and L-type Ca^2+^ current inhibition. *P* < 0.05 was considered to be statistically significant (marked as ^∗^) and the higher significance level was set at *P* < 0.01 (marked as ^∗∗^).

## Results

### DS-201 Relaxes the Endothelium-Denuded Artery Rings Pre-constricted by PE and High K^+^

**FIGURE 1:**
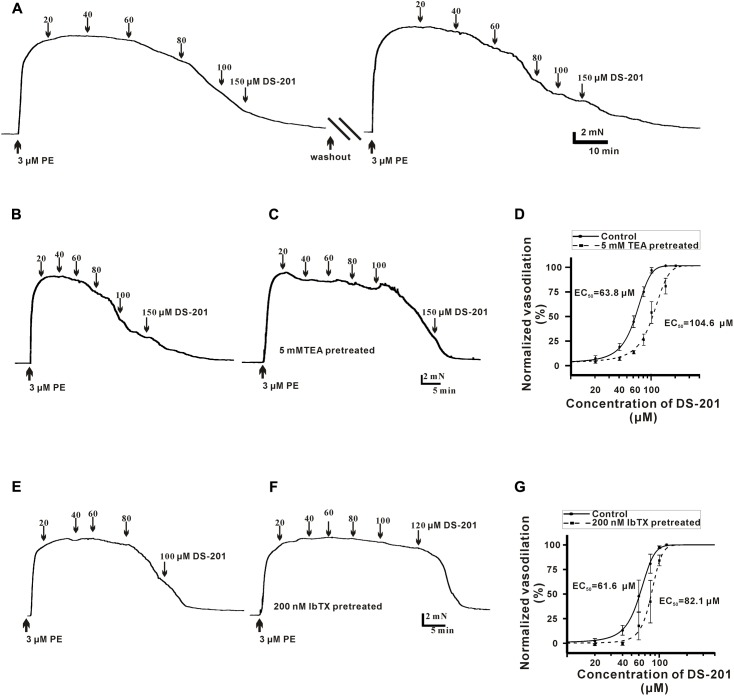
Effect of DS-201 on the vasodilation with or without TEA or IbTX treatment in the PE-preconstricted artery rings. **(A)** A typical recording shows that DS-201 relaxed the PE-preconstricted artery rings in a concentration-dependent manner and the effect was reversible after 1 h duration of washout and balance. **(B)** Traces were from one representative artery ring to show the effect of 20–150 μM DS-201on vasorelaxation. **(C)** TEA (5 mM) treatment reduced the vasodilation of DS-201in a representative PE-preconstricted artery ring. **(D)** Concentration–response curves fitted with Hill function showed that TEA (5 mM) shifted the curve rightward. The data were obtained with seven artery rings from six rats for each group. Hill function: Inhibition % = *X*^n^/(*k*^n^+*X*^n^), *X* as the concentration of DS-201, *K* as the Michaelis constant (i.e., EC50), and *n* as the Cooperative sites. **(E)** A typical recording shows that DS-201 relaxed the representative PE-preconstricted artery ring in a concentration-dependent manner. **(F)** IbTX (200 nM) reduced the vasodilation of DS-201in a representative PE-preconstricted artery ring and **(G)** Concentration -response curves fitted with Hill function and the data show that IbTX shifted the curve rightward. The data were obtained with four artery rings from four rats for each group.

**FIGURE 2:**
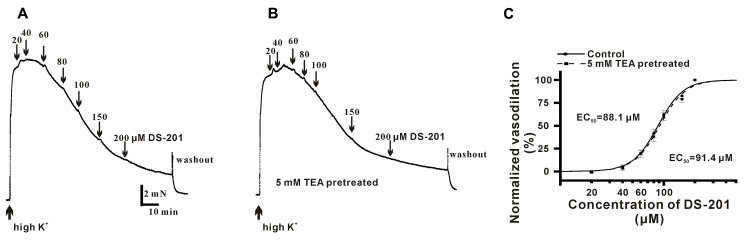
Effect of DS-201 (20–200 μM) on vasodilation in high K^+^-preconstricted artery rings. **(A)** The typical recordings show that DS-201 relaxed high K^+^-preconstricted artery rings in a concentration-dependent manner. **(B)** The typical recordings show that TEA (5 mM) did not affect the effect of DS-201 on vasodilation and **(C)** Concentration-response curves show that TEA did not shift the curve. The data were obtained with eight artery rings from six rats.

To measure the direct effect of DS-201 on vasorelaxation in the VSMCs, the endothelium layer of artery rings were denuded by perfusion of 0.1% triton solution before the measurement and only the arteries with less than 10% relaxation induced by 1 μM ACh were used for experiments. The artery rings were pre-constricted with 3 μM PE, and various concentrations of DS-201 (20, 40, 60, 80, 100, and 150 μM) were added into the bath solution when the artery rings were fully equilibrated. A typical tension recording is shown in **Figure 1** and the results showed that DS-201 relaxed the PE-preconstricted artery rings in a concentration-dependent manner and the effect was reversible (**Figure 1A**). To investigate the role of K^+^ channel, artery rings were incubated with 5 mM TEA to block K^+^ channel (**Figures 1B–D**). The data in **Figure 1D** showed that the concentration-response curve of DS-201 was shifted rightward after the blockade of K^+^ channel by TEA. The EC_50_ of DS-201 was changed from 64.2 ± 2.8 to 107.4 ± 8.6 μM (*p* < 0.01). Furthermore, the role of BK_Ca_ channel was also investigated for the vasorelaxing effect of DS-201 with a selective BK_Ca_ channel blocker IbTX (200 nM, **Figures 1E–G**). Results showed that IbTX also shifted the concentration–response curve of DS-201 to a rightward (**Figure 1G**). The EC_50_ of DS-201 was changed from 62.2 ± 6.3 to 81.0 ± 8.4 μM. However, TEA could not shift the concentration-response curve of DS-201 at the same condition after the artery rings were pre-constricted in 60 mM high K^+^ solution (**Figure 2**). The EC_50_ of DS-201 on vasorelaxation was 92.1 ± 5.5 and 88.8 ± 4.2 μM, respectively, with or without TEA treatment (*p* > 0.05). These results indicate that the effect of DS-201 on vasorelaxation was not solely due to its effect on K^+^ channel because blockade of K^+^ channel did not completely affect its vasorelaxing effect in the precontracted artery rings by PE or high K^+^ solution, implying that an alternative mechanism may be existed for the effect of DS-201 on vascular tension relaxation.

### DS-201 Alleviates Depolarization-Induced Ca^2+^ Transients in the VSMCs of Rats

**FIGURE 3:**
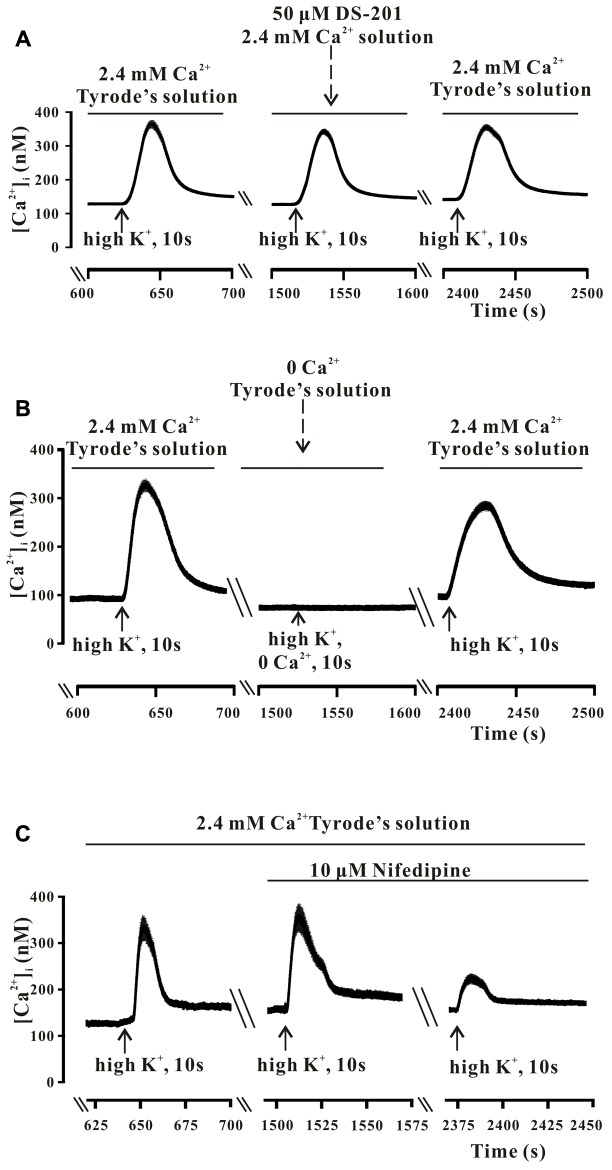
The high K^+^-induced intracellular Ca^2+^ transients in the mesenteric VSMCs of rats. **(A)** The typical recordings show that high K^+^ (60 mM) did not affect the high K^+^-induced intracellular Ca^2+^ transients. The data were obtained from 13 cells in one measurement. **(B)** The typical recordings show that high K^+^ could induce Ca^2+^ transient only in the presence of extracellular Ca^2+^. The data were obtained from 28 cells in one measurement. **(C)** The typical recordings show that the high K^+^-induced intracellular Ca^2+^ transients were sensitive to the L-type Ca^2+^ channel blocker, Nifedipine (10 μM). The data were obtained from 18 cells in one measurement.

**FIGURE 4:**
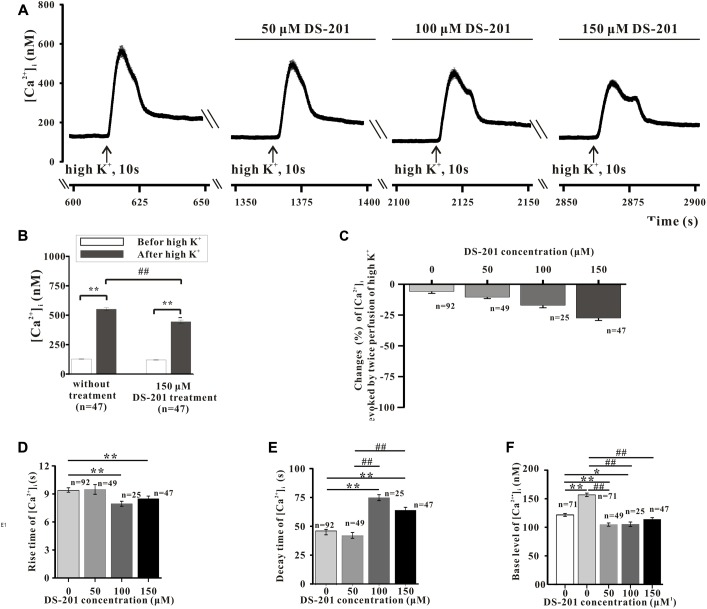
Effect of DS-201 on high K^+^-induced intracellular Ca^2+^ transients in the mesenteric VSMCs of rats. **(A)** The typical recordings show that the inhibitory effects of DS-201 (50, 100, and 150 μM) on the Ca^2+^ transients induced by high K^+^ was concentration- dependent. The data were obtained with13 cells in one measurement. **(B)** The effect of 150 μM DS-201 on high K^+^-induced changes of intracellular Ca^2+^ before or after treatment of 60 mM High K^+^ solution for 10 sec. ^∗∗^*p* < 0.01, between the groups of before and after high K^+^ solution; ##*p* < 0.01 between the groups of no treatment and DS-201 treatment. The data were obtained with 47 cells from five rats for each group. **(C)** Concentration-response of DS-201 on high K^+^-induced intracellular Ca^2+^ transients. **(D)** The rise time of high K^+^-induced intracellular Ca^2+^ transients. **(E)** The decay time of high K^+^ -induced intracellular Ca^2+^ transients and **(F)** the resting [Ca^2+^]_i_ levels with different concentrations of DS-201. Note that DS-201 inhibited the increase of base [Ca^2+^]_i_ level induced by high K^+^. For **C–F**: the cells used in the study were labeled at the relative bar from five rats. The *post hoc* comparison was analyzed with one-way ANOVA followed by student-Newman–Keuls test.

Freshly isolated mesenteric VSMCs were used to measure the Ca^2+^ transients. The typical recordings of high K^+^-induced Ca^2+^ transients and the effect of DS-201are shown in **Figures 3**, **4**, respectively. The data reveal that high K^+^ solution did not affect Ca^2+^ response to the VSMCs (**Figure 3A**), high K^+^-induced Ca^2+^ transients was not induced in the VSMCs incubated in Ca^2+^-free Tyrode’s solution with 0.2 mM EGTA (**Figure 3B**), but was inhibited by the L-type Ca^2+^ channel inhibitor, nifedipine (10 μM, **Figure 3C**). The result indicated that high K^+^-induced Ca^2+^ transients were mainly induced due to the effect of extracellular Ca^2+^ influx. Pre-incubation of DS-201 for 10 min decreased Ca^2+^ response in the VSMCs in high K^+^ solution in a concentration-dependent manner. As shown in **Figure 4A**, DS-201 slightly decreased Ca^2+^ response to high K^+^ at lower concentrations (50 and 100 μM) but significantly decreased the response at higher concentration (150 μM). Furthermore, [Ca^2+^]_i_ was changed from the base level of 127.4 ± 4.2 to the peak of 551.1 ± 12.6 nM in control group and 120.0 ± 4.0 to 444.3 ± 14.0 nM in 150 μM DS-201 treated group in high K^+^ solution (**Figure 4B**). DS-201 at 50, 100 and 150 μM decreased Ca^2+^ response to high K^+^ by 10.5 ± 1.1%, 17.1 ± 2.0%, and 27.4 ± 2.0%, respectively (**Figures 4C**). The results showed that the rise time of Ca^2+^ transients became shorter and the decay time became longer when the concentrations of DS-201 were higher than 100 μM (**Figures 4D,E**). DS-201 also decreased the base level of [Ca^2+^]_i_ (**Figure 4F**). These results suggest that DS-201 did not obviously affect the [Ca^2+^]_i_ level at the lower concentrations, whereas remarkably suppressed the [Ca^2+^]_i_ transient at higher concentrations in the VSMCs. In addition, DS-201 also affected the time for Ca^2+^ reaching to the peak and removal from the cytoplasm.

### DS-201 Inhibits L-Type Ca^2+^ Channel

**FIGURE 5:**
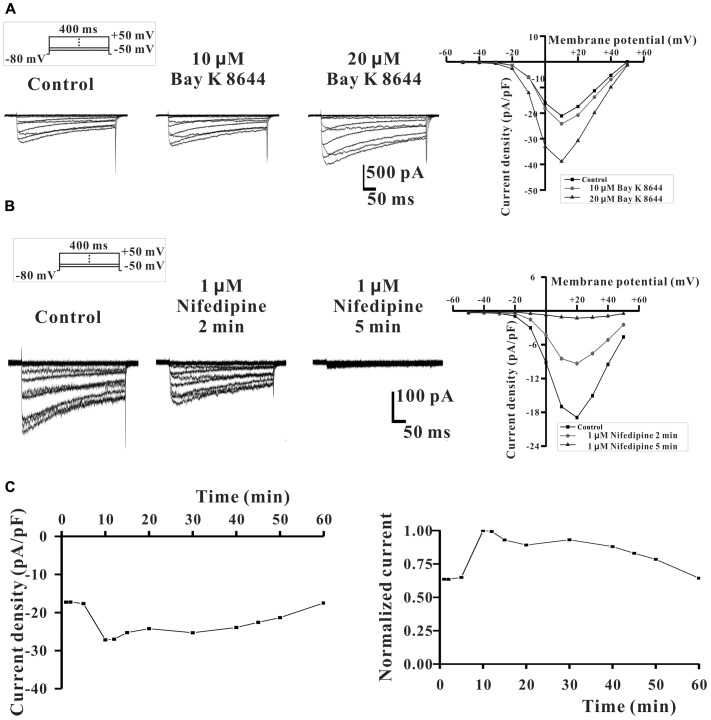
The expression of L-type Ca^2+^ channel in L_Ca_ channel-transfected HEK293 cells. **(A)** The typical recordings show that L_Ca_ channel was activated by 10 and 20 μM Bay K 8644 (an agonist of L_Ca_ channel). **(B)** L_Ca_ channel was inhibited by 1 μM nifedipine (an antagonist of L_Ca_ channel). **(C)** *I*_Ca,__L_ was relative stable and *I*_Ca,L_ rundown was less than 10% within 15–40 min after the whole-cell configuration formation.

**FIGURE 6:**
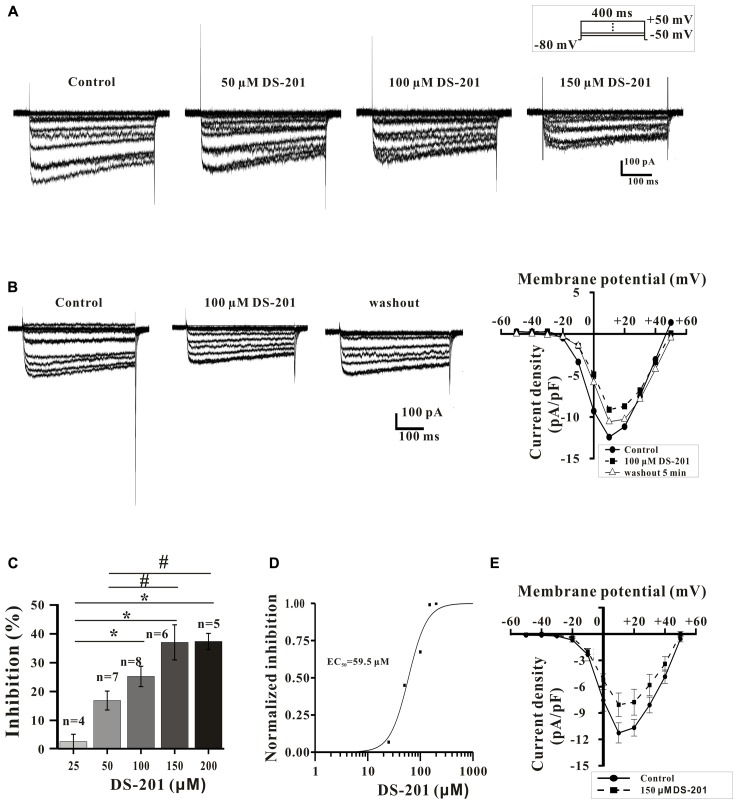
Effect of DS-201 on L-type Ca^2+^ channel in L_Ca_ channel-transfected HEK293 cells. **(A)** *I*_Ca,L_ was inhibited by DS-201 (50, 100, and 150 μM) in a concentration-dependent manner. **(B)** The inhibition of *I*_Ca,L_ induced by DS-201(100 μM) was partially reversible. **(C)** Histograms show that the inhibition of peak Ca^2+^ currents by DS-201 (25–200 μM) was concentration dependent measured at +10 mV membrane potential. **(D)** Concentration-response curves of DS-201 fitted with Hill function. The Data were panel **C**. Hill function: [Inhibition % = *X*^n^/(*k*^n^+*X*^n^)], *X* as the concentration of DS-201, *K* as the Michaelis constant (i.e., EC50), and *n* as the Cooperative sites. **(E)** Effect of DS-201 on the I-V relationship of *I*_Ca,L_. Data obtained from 6 cells showing that 150 μM DS-201 inhibited the peak current, but did not change the relationship including maximum activated potential and reversal potential. The data were obtained from the average of eight experiments with 4–8 cells in one group.

We also further studied the direct inhibitory effect of DS-201 on the activity of L_Ca_ channel in L_Ca_ channel-transfected HEK293 cells. The data in **Figure 5A** displayed the typical traces of *I*_Ca,L_ and the I-V curve, and showed that the *I*_Ca,L_ was elicited by Bay K 8644 (10 and 20 μM), a agonist of L-type Ca^2+^ channel, indicating that the recording of *I*_Ca,L_ was correct. The data in **Figure 5B** displayed the typical *I*_Ca,L_ traces and the I-V curve, and show that *I*_Ca,L_ was inhibited by nifedipine (1 μM), an antagonist of L-type Ca^2+^ channel. The data in **Figure 5C** showed that the *I*_Ca,L_ was stable formation in whole-cell configuration (rundown <10%) within 15–40 min. These channel properties were consistent with those of L-type Ca^2+^ channel in SMCs reported previously (Li et al., 2013). The data in **Figure 6A** show that the inhibition of DS-201 on *I*_Ca,L_ was concentration-dependent in the typical recordings. DS-201 at the concentrations of 25, 50, 100, 150, and 200 μM decreased the *I*_Ca,L_ by 2.5 ± 2.5% (*n* = 4), 16.7 ± 3.3% (*n* = 7), 25.1 ± 3.5% (*n* = 8), 36.9 ± 6.1% (*n* = 6), and 37.2 ± 2.8% (*n* = 5), respectively (**Figure 6C**). The normalized inhibition against DS-201 concentration and the Hill fits with the EC_50_ of DS-201 on L-type Ca^2+^ channel was 59.5 μM, the cooperative sites were 2.5 (**Figure 6D**). The I–V relationship of DS-201 at 150 μM was illustrated and showed that the relationship including maximum activated potential and reversal potential has no significant changes compared to the control (**Figure 6E**). The result demonstrated that DS-201 was an inhibitor of L-type Ca^2+^ channel and the inhibition of L-type Ca^2+^ channel may contribute to the vasorelaxing effect of DS-201.

**FIGURE 7:**
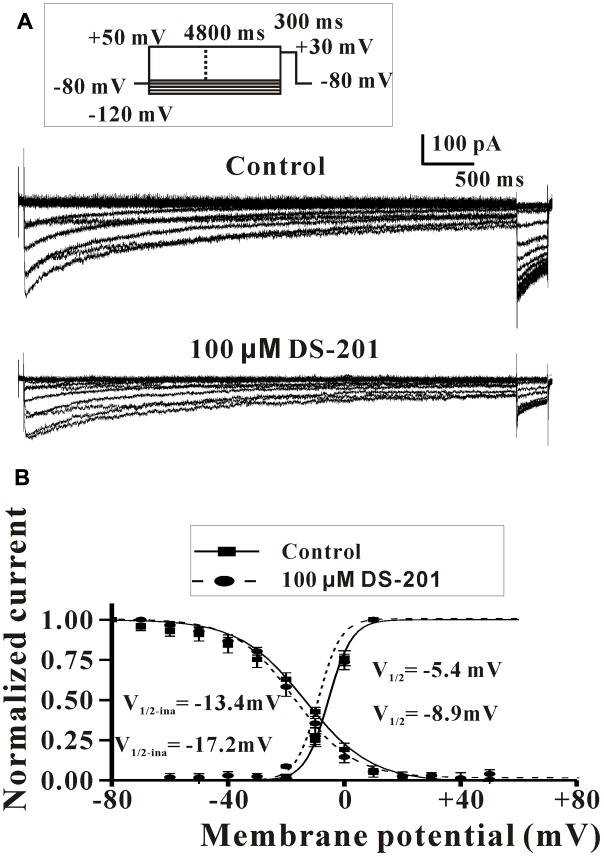
Effect of DS-201 on the activation and inactivation of L-type Ca^2+^ channel in L_Ca_ channel-transfected HEK293 cells. **(A)** Typical recordings show that the activation and inactivation of L-type Ca^2+^ channel under membrane potentials of -100 to +30 mV with or without DS-201 (100 μM) treatment. **(B)** For the activation curve, I–V relations were obtained from the variables of normalized current (*I*_peak_/*I*_peak-max_) of *I*_Ca,L_ and the relative stimulation voltage. For the inactivation curve, I–V relations were obtained from the variables of normalized current (*I*_peak_/*I*_peak-max_) of *I*_Ca,L_ at tested potential of +30 mV and the relative voltage of conditioning prepulses. I–V curves were fitted to the Boltzmann function: *y* = 1/(1 + exp ([V-V_1/2_)/k]), where V as the tested membrane potential, *V*_1/2_ as the potential of half-maximal activation, and *k* as the slope factor. The data were obtained from three cells of three experiments.

**FIGURE 8:**
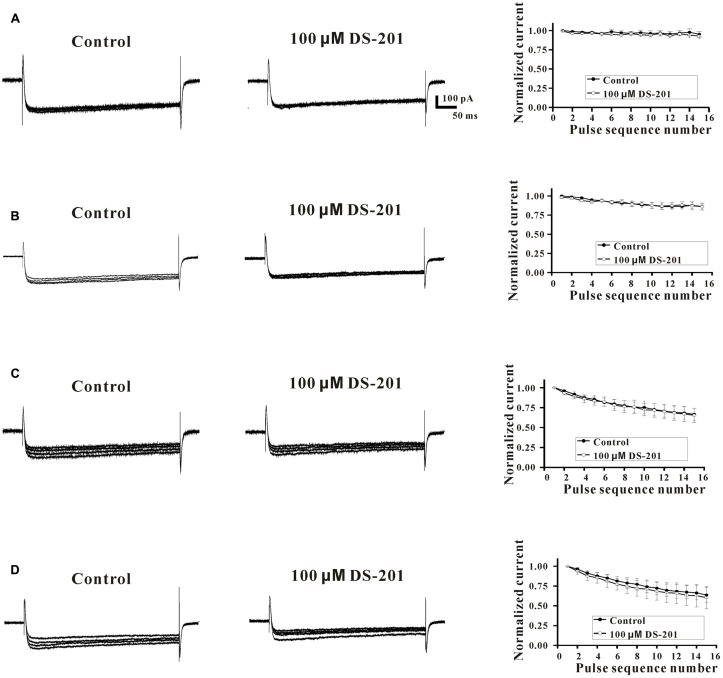
Effect of DS-201 on L-type Ca^2+^ channel was rate-independent in L_Ca_ channel-transfected HEK293 cells. Superimposed typical tracings (pulse sequence number: 1, 5, 10, 15) of *I*_Ca,L_ were obtained at 0.1 **(A)**, 0.2 **(B)**, 0.7 **(C)**, and 2.0 Hz **(D)**, at –80 mV to +10 mV membrane potential in the absent or presence of 100 μM DS-201. The normalized peak currents of *I*_Ca,L_ (I_peak_/I_peak-first pulse_) were plotted against each pulse sequence number. The data were obtained from five cells of five experiments.

Next, we further examined the potential effects of DS-201 on the activation and inactivation kinetics of L-type Ca^2+^ channel and rate-dependent effect in L_Ca_ channel-transfected HEK293 cells (**Figures 7**, **8**). The results showed that, both of half-activation and half-inactivation voltages of *I*_Ca,L_ were only slightly shifted leftward after treatment of 100 μM DS-201, from - 5.4 to - 8.9 mV(*p* > 0.05), and -13.4 to -17.2 mV(*p* > 0.05), respectively (**Figure 7**). The rate-dependent effect of DS-201 (100 μM) on *I*_Ca,L_ in the cells was investigated at 0.1, 0.2, 0.7, and 2.0 Hz stimulated pulses (**Figure 8**). The peak amplitude of *I*_Ca,L_ was not changed at 0.1 and 0.2 Hz but changed at 0.7 and 2.0 Hz after 15 repetitive depolarizing pulses. Increase of the frequency of stimuli induced a progressive decline of the *I*_Ca,L_ amplitude at 0.7 and 2.0 Hz depolarization when the holding potential clamped to +10 mV. However, DS-201 (100 μM) did not change the suppression of *I*_Ca,L_ induced by increased stimuli frequency. These results suggest that DS-201 has rate-independent blockage on L-type Ca^2+^ channel with minimal effects on the activation and inactivation kinetics of the channel.

## Discussion

The present study was aimed to investigate the new mechanisms underlying the beneficial effects of DS-201 on cardiovascular system. The results demonstrated for the first time that the effect of DS-201 on vasorelaxation was via inhibiting the L-type Ca^2+^ channel. Furthermore, we also demonstrated that DS-201 could affect Ca^2+^ influx and L-type Ca^2+^ channel in a rate-independent manner in the VSMCs of rats.

Calcium mobilization is a key upstream signal in the activity of SMCs. Similar to other SMCs, VSMCs need Ca^2+^ influx to initiate constriction. Change of [Ca^2+^]_i_ may result in dynamic equilibrium of transmembrane transport, ER uptake and release of Ca^2+^. It is well known that Ca^2+^ transient is important in the process of excitation–contraction (E–C) coupling of VSMCs. Because of the high expression level in the VSMCs, L-type calcium channel has the greatest influence on global [Ca^2+^]_i_, and its activity largely determines the contractile state of ASMCs and ultimately the vessel diameter (Knot and Nelson, 1998). However, the intracellular Ca^2+^ level is not the only determining factor for the contractile state of ASMCs. BK_Ca_ channel is sensitive to intracellular Ca^2+^. Any direct or indirect perturbation of [Ca^2+^]_i_ may not only result in the changes of the E-C coupling but also the buffering mechanism of BK_Ca_ channel. It is reasonable to believe that the agents altering the activities of Ca^2+^ channel or BK_Ca_ channel may affect vascular tone.

We previously reported that the vasorelaxing effect of DS-201 was associated with the activation of BK_Ca_ channel (Yang et al., 2008). DS-201 (20–150 μM) increased BK_Ca_ currents by 5.4–173.2 fold in an almost linear shape in the inside-out patches. However, DS-201 induced the change of BK_Ca_ currents in a bell-shaped under the whole-cell configuration (Yang et al., 2008). This difference between single channel patch clamp and whole-cell configuration suggests that some other factor(s) may be involved in the action of DS-201 on BK_Ca_ channel in the cells. Therefore, we supposed that the factor may be involved with Ca^2+^ channel. In other words, DS-201 may also exhibit an (inhibitory) effect on Ca^2+^ channel.

Ca^2+^-mediated increase of contractility in the VSMC may be the possible target for vasorelaxation of drugs. It is well established that vascular tone can be increased by the activation of myosin with myosin light chain kinase (MLCK). MLCK is a Ca/CaM-dependent kinase and activated by increases the level of [Ca^2+^]_i_ in the cytoplasm of cells (Brozovich et al., 2016). To investigate the effect of DS-201 on the blood vessel and influence of [Ca^2+^]_i_, we firstly studied the vasorelaxation of the endothelium-denuded artery rings pre-constricted by PE and high K^+^. Our results showed that the blockade of K^+^ channel by TEA and IbTX could not totally block the vasorelaxing effect of DS-201 in PE- precontrated artery rings, thus an alternative vasorelaxing mechanism of DS-201 may be existed in addition to the activation of K^+^ channel. The present study with the artery rings precontracted by high K^+^ solution showed that TEA treatment did not affect the relaxing effect of DS-201, indicating that K^+^ channel was not involved for the effect. May be mainly due to the opening of Ca^2+^ channel with higher membrane potential in high K^+^ condition, the K^+^ channel effect of DS-201 on counteracting membrane depolarization could not be realized. PE is a α receptor agonist, one of the G-protein coupled receptor-mediated agonists, and high K^+^ induces depolarization of cell membrane via increase of [Ca^2+^]_i_, so [Ca^2+^]_i_ can be increased by these two approaches. Therefore, our results demonstrated that the effect of DS-201 on the relaxation of the VSMCs is related to [Ca^2+^]_i_ in the artery rings. In order to validate the hypothesis, we further investigated the effect of DS-201on the suppression of [Ca^2+^]_i_ transient in the VSMCs of rats. The data showed that DS-201 affected Ca^2+^ transient including the base level, amplitude, and kinetics of [Ca^2+^]_i_ (**Figures 3**, **4**). The studies confirm that inhibition of Ca^2+^ influx in the VSMCs is important for the vasorelaxation effect of DS-201. Our results are consistent with the reports by Lam et al. (Lam et al., 2006, 2008). They have also shown that Danshen and its fraction of a lipophilic component relaxed arteries through the inhibition of calcium channel. Therefore, the regulation of intracellular Ca^2+^ by DS-201 may play an important role in the vasorelaxation, and certain concentrations of DS-201 may determine its efficacy on BK_Ca_ and Ca^2+^ channels.

In addition, we also studied the effect of DS-201 on L-type Ca^2+^ current in L_Ca_ channel-transfected HEK-293 cells (**Figures 5**–**8**). Our results demonstrated that DS-201 did not affect the suppression of I_Ca,L_ induced by increased stimuli frequency open channel blockade, suggesting that it was a rate-independent blockage on L-type Ca^2+^ channel and ruled out open channel blockade by DS-201. DS-201 may either interact with open state or inactivated state, perhaps due to slowing down the recovery from inactivation to execute its effect. We will perform additional studies to confirm this hypothesis in the future. The advantage for use of the cell model with simple channel expression is that the cells can avoid the interaction between the channels to produce complex results because BK_Ca_ channel and Ca^2+^ channel are closely associated. We demonstrated that DS-201 was a Ca^2+^ channel inhibitor and the effect was concentration-dependent. Therefore, we discovered an alternative mechanism underlying the vasorelaxing effect of DS-201.

Danshen is a commonly used traditional Chinese medicinal herb, and numbers of studies have been carried out to elucidate the mechanisms (Cheng, 2007; Kim et al., 2007; Morton et al., 2015; Yu et al., 2016). Here we for the first time found a novel mechanism underlying the vasorelaxing effect of DS-201 (a main active derivative of Danshen), i.e., DS-201 inhibited L-type Ca^2+^ channel and modulated intracellular Ca^2+^ level through complex effects on K^+^ and Ca^2+^ channels, and finally reduced the vascular tension. Our findings may provide better understanding of the cardiovascular action of DS-201 and favor the use of DS-201 and/or Danshen in the treatment of cardiovascular diseases clinically. However, the limitation of our study is that we evaluated the effects of DS-201 in transfected HEK293 cells and the cells may not precisely reflect the actions of DS-201 on native L-type channel in VSMCs. The difference may exist between transfected HEK293 cells and VSMCs for the action of DS-201 on L-type channel because multiple factors could influence drug action including other proteins and cellular factors affecting the binding of the drug to the channel, the half-life and/or distribution of drug in VSMCs and so on.

## Declaration Of Transparency And Scientific Rigor

This Declaration acknowledges that this paper adheres to the principles for transparent reporting and scientific rigor of preclinical research recommended by funding agencies, publishers and other organizations engaged with supporting research.

## Author Contributions

X-DZ and C-XH designed the studies, performed the experiments, acquired and analyzed the data, and drafted the manuscript. JC, JW, and P-YL carried out the measurements of arterial tension and [Ca^2+^]_i_. NW and GL carried out the patch clamp experiments. X-RZ and J-MC participated in the protocol design and critically revised the manuscript. YY designed and directed the protocol and wrote and critically revised the manuscript.

## Conflict of Interest Statement

The authors declare that the research was conducted in the absence of any commercial or financial relationships that could be construed as a potential conflict of interest.

## Abbreviations

ACh: acetylcholine
BK_Ca_ channel: large conductance Ca^2+^-activated K^+^ channel
DS-201: Sodium tanshinone II-A sulfonate
EC_50_: half maximal effective concentration
IbTX: Iberiotoxin
*I*_Ca,L_: L-type Ca^2+^ current
PE: phenylephrine
STOCs: spontaneous transient outward K^+^ currents
TEA: tetraethylammonium
VSMCs: vascular smooth muscle cells

## References

[B1] Brozovich F. V. Nicholson C. J. Degen C. V. Gao Y. Z. Aggarwal M. Morgan K. G. (2016). Mechanisms of vascular smooth umuscle contraction and the basis for pharmacologic treatment of smooth muscle disorders. *Pharmacol. Rev.* 68 476–532. 10.1124/pr.115.010652 27037223 PMC4819215

[B2] Chan P. Liu I. M. Li Y. X. Yu W. J. Cheng J. T. (2009). Antihypertension induced by tanshinone IIA isolated from the roots of *Salvia miltiorrhiza*. *Evid. Based Complement. Alternat. Med.* 2011 :392627. 10.1093/ecam/nep056 19542183 PMC3135424

[B3] Cheng T. O. (2006). Danshen: a popular Chinese cardiac herbal drug. *J. Am. Coll. Cardiol.* 47 1498; author reply 1499–1500. 10.1016/j.jacc.2006.01.001 16580549

[B4] Cheng T. O. (2007). Cardiovascular effects of Danshen. *Int. J. Cardiol.* 121 9–22. 10.1016/j.ijcard.2007.01.004 17363091

[B5] Curtis M. J. Bond R. A. Spina D. Ahluwalia A. Alexander S. P. Giembycz M. A. (2015). Experimental design and analysis and their reporting: new guidance for publication in BJP. *Br. J. Pharmacol.* 172 3461–3471. 10.1111/bph.12856 26114403 PMC4507152

[B6] Grynkiewicz G. Poenie M. Tsien R. Y. (1985). A new generation of Ca^2+^ indicators with greatly improved fluorescence properties. *J. Biol. Chem.* 260 3440–3450. 3838314

[B7] Jaggar J. H. Porter V. A. Lederer W. J. Nelson M. T. (2000). Calcium sparks in smooth muscle. *Am. J. Physiol. Cell Physiol.* 278 C235–C256. 10.1152/ajpcell.2000.278.2.C235 10666018

[B8] Kim D. D. Sanchez F. A. Duran R. G. Kanetaka T. Duran W. N. (2007). Endothelial nitric oxide synthase is a molecular vascular target for the Chinese herb Danshen in hypertension. *Am. J. Physiol. Heart Circ. Physiol.* 292 H2131–H2137. 10.1152/ajpheart.01027.2006 17172272

[B9] Knot H. J. Nelson M. T. (1998). Regulation of arterial diameter and wall [Ca^2+^] in cerebral arteries of rat by membrane potential and intravascular pressure. *J. Physiol.* 508 199–209. 10.1111/j.1469-7793.1998.199br.x 9490839 PMC2230857

[B10] Lam F. F. Yeung J. H. Chan K. M. Or P. M. (2008). Dihydrotanshinone, a lipophilic component of *Salvia miltiorrhiza* (danshen), relaxes rat coronary artery by inhibition of calcium channels. *J. Ethnopharmacol.* 119 318–321. 10.1016/j.jep.2008.07.011 18682284

[B11] Lam F. F. Yeung J. H. Cheung J. H. Or P. M. (2006). Pharmacological evidence for calcium channel inhibition by danshen (*Salvia miltiorrhiza*) on rat isolated femoral artery. *J. Cardiovasc. Pharmacol.* 47 139–145. 10.1097/01.fjc.0000197540.12685.ce 16424798

[B12] Li P. Y. Zeng X. R. Cheng J. Wen J. Inoue I. Yang Y. (2013). Rhynchophylline-induced vasodilation in human mesenteric artery is mainly due to blockage of L-type calcium channels in vascular smooth muscle cells. *Naunyn Schmiedebergs Arch. Pharmacol.* 386 973–982. 10.1007/s00210-013-0888-6 23812676

[B13] Morton J. S. Andersson I. J. Cheung P. Y. Baker P. Davidge S. T. (2015). The vascular effects of sodium tanshinone IIA sulphonate in rodent and human pregnancy. *PLOS ONE* 10:e0121897. 10.1371/journal.pone.0121897 25811628 PMC4374693

[B14] Tan X. Yang Y. Cheng J. Li P. Inoue I. Zeng X. (2011). Unique action of sodium tanshinone II-A sulfonate (DS-201) on the Ca(^2+^) dependent BK(_Ca_) activation in mouse cerebral arterial smooth muscle cells. *Eur. J. Pharmacol.* 656 27–32. 10.1016/j.ejphar.2011.01.028 21284944

[B15] Valli G. Giardina E. G. (2002). Benefits, adverse effects and drug interactions of herbal therapies with cardiovascular effects. *J. Am. Coll. Cardiol.* 39 1083–1095. 10.1016/S0735-1097(02)01749-7 11923030

[B16] Wang Y. X. Zheng Y. M. Abdullaev I. Kotlikoff M. I. (2003). Metabolic inhibition with cyanide induces calcium release in pulmonary artery myocytes and *Xenopus* oocytes. *Am. J. Physiol. Cell Physiol.* 284 C378–C388. 10.1152/ajpcell.00260.2002 12388060

[B17] Wray S. Burdyga T. Noble K. (2005). Calcium signalling in smooth muscle. *Cell Calcium* 38 397–407. 10.1016/j.ceca.2005.06.018 16137762

[B18] Yang Y. Cai F. Li P. Y. Li M. L. Chen J. Chen G. L. (2008). Activation of high conductance Ca^(2+^)-activated K^(+^) channels by sodium tanshinoneII-A sulfonate (DS-201) in porcine coronary artery smooth muscle cells. *Eur. J. Pharmacol.* 598 9–15. 10.1016/j.ejphar.2008.09.013 18831973

[B19] Yang Y. Li P. Y. Cheng J. Cai F. Lei M. Tan X. Q. (2013). IP3 decreases coronary artery tone via activating the BKCa channel of coronary artery smooth muscle cells in pigs. *Biochem. Biophys. Res. Commun.* 439 363–368. 10.1016/j.bbrc.2013.08.079 24012825

[B20] Yu L. Cheng J. Huang W.-J. Tan X.-Q. Mao L. Liu Z.-F. (2016). Liposome intracellular delivery of *Salvia miltiorrhiza* Bge. deprivative DS-201 improves its BKCa channel-activating and vasorelaxing effect. *Sciences Sci. Bulletin Bull.* 61 622–631. 10.1007/s11434-016-1046-6

[B21] Zhou L. Zuo Z. Chow M. S. (2005). Danshen: an overview of its chemistry, pharmacology, pharmacokinetics, and clinical use. *J. Clin. Pharmacol.* 45 1345–1359. 10.1177/0091270005282630 16291709

